# Editorial: Nutritional management of patients with inborn errors of metabolism

**DOI:** 10.3389/fnut.2024.1523957

**Published:** 2024-11-28

**Authors:** Consuelo Pedrón-Giner, Luis Aldamiz-Echevarria, María L. Couce

**Affiliations:** ^1^Department of Pediatrics, Niño Jesús University Children's Hospital, Madrid, Spain; ^2^Instituto de Investigación Sanitaria de Santiago de Compostela (IDIS), Santiago de Compostela, Spain; ^3^University Clinical Hospital of Santiago de Compostela, RICORS-SAMID, Santiago de Compostela, Spain

**Keywords:** Inborn errors of metabolism, nutritional therapy, dietary carbohydrate, dietary lipid, dietary protein, energy requirement

Inborn Errors of Metabolism (IEM) are defined as biochemical alterations of genetic origin caused by a specific defect in the structure and function of a protein. Their main characteristic is their heterogeneity in terms of causes, clinical expression, diagnostic methods and treatments (1–5). Another characteristic fact is that it includes a large number of diseases and that each of them has a very low frequency of presentation, although, globally assessed, they can affect 1:500 live newborns (6). Until a few years ago, most of them were lethal or had serious sequelae. Nowadays, although many of them are life-threatening, more and more treatments are becoming available, which are not curative but allow an acceptable quality of life (1, 2).

The IEM that interferes, among others, with the metabolism of proteins, fats and carbohydrates, can be divided from the pathophysiological point of view into 3 groups (7): (1) alterations in intermediary metabolism, (2) primary affectations in energy metabolism and (3) disorders in the metabolism of complex molecules and channelopathies. Nutritional and dietary treatment is the mainstay of the global management of IEM affecting intermediary metabolism and some energy metabolism, to keep the disease under control, but it can also allow other patients to receive their specific treatment in the best possible conditions (2, 8).

Accurate dietary management of protein, lipid and carbohydrate metabolism in these patients is essential to avoid metabolic decompensation, which can have serious multi-organ consequences, especially neurological, and even death. Dietary adherence should be sought. At the same time, the diets prescribed for IEM must be personalized, taking into account the greater or lesser severity of the disease, the patient's clinical status, tolerance, age and neurodevelopment. However, despite the advances of recent years, this dietary and nutritional therapy is sometimes very complex and difficult to comply with, requiring a strict evolutionary control that is not always well known (2, 8).

Therefore, the nutritional treatment of IEM is an extraordinary challenge, and the aim of this Research Topic was to collect any kind of scientific article exploring any of these metabolic diseases showing their peculiar clinical or diagnostic features, and the possible advances in their dietary treatment, thus adding to the kaleidoscope of reality. The result is truly diverse showing a range of diseases and a multiplicity of approaches.

Firstly, the type of ketogenic diet (9) applied to 13 children with glucose transport deficiency type 1 and its impact on the lipid and liver profile is analyzed. Dietary recommendations are given, and it is shown that despite the high fat intake, there are no alterations in the biochemical parameters analyzed, which may help doctors and patients to recommend and follow this treatment without fear, although well monitored (Salazar et al.).

The following is a systematic review of the therapeutic approach in Latin America for one of the most prevalent diseases in this group, phenylketonuria (PKU). Challenges in this geographical setting include health gaps, resource scarcity and dependence on international guidance (10). This study highlights the urgent need for collaborative efforts between healthcare institutions, policy makers and international organizations to close the gaps in PKU management in Latin America (Aguirre et al.). This involves multifaceted approaches and critical multidisciplinary teams that ensure the best care for each patient.

The basis of dietary treatment of immune disorders, a very rare and diverse group of pathologies (11), is then reviewed in order to establish minimum standards. This review highlights the scarcity of studies evaluating dietary intake, anthropometry and nutritional biochemistry in these patients, with considerable heterogeneity between studies (Freer et al.). This fact does not surprise us at all since we know that significant awareness is needed among professionals regarding the complete nutritional assessment of their patients with validated and consistent tools.

Finally, the Research Topic closes with a clinical case of ornithine transcarbamylase deficiency, a urea cycle disorder (12). The therapeutic approach with protein restriction and ammonia sequestration was started at birth due to its prenatal diagnosis. The rigor of nutritional control during its evolution shows a good evolution, but many aspects remain to be discussed (Martín-Rivada et al.).

We would like to thank our contributors and hope that Research Topic will be to the liking of our readers and useful for their practice.
